# Promising Scaffold-Free Approaches in Translational Dentistry

**DOI:** 10.3390/ijerph17093001

**Published:** 2020-04-26

**Authors:** Marco Tatullo, Benedetta Marrelli, Francesca Palmieri, Massimiliano Amantea, Manuel Nuzzolese, Rosa Valletta, Barbara Zavan, Danila De Vito

**Affiliations:** 1 Department of Basic Medical Sciences, Neurosciences and Sense Organs, University of Bari, 70122 Bari, Italy; danila.devito@uniba.it; 2 Marrelli Health, Tecnologica Research Institute, 88900 Crotone, Italy; benedetta.marrelli@calabrodental.it (B.M.); francesca.palmieri@calabrodental.it (F.P.); massimiliano.amantea@calabrodental.it (M.A.); 3 Department of Therapeutic Dentistry, Sechenov University Russia, Moscow 119146, Russia; 4 North Manchester General Hospital, Manchester M8 5RB, UK; manuelnuzzolese@libero.it; 5 Department of Neurosciences, Reproductive and Odontostomatological Sciences, University of Naples, 80131 Naples, Italy; rosa.valletta@unina.it; 6 Department of Medical Sciences, University of Ferrara, 44121 Ferrara, Italy; barbara.zavan@unife.it

**Keywords:** scaffolds, dental materials, regenerative dentistry, dentistry

## Abstract

Regenerative medicine has recently improved the principal therapies in several medical fields. In the past ten years, the continuous search for novel approaches to treat the most common dental pathologies has developed a new branch called regenerative dentistry. The main research fields of translational dentistry involve biomimetic materials, orally derived stem cells, and tissue engineering to populate scaffolds with autologous stem cells and bioactive growth factors. The scientific literature has reported two main research trends in regenerative dentistry: scaffold-based and scaffold-free approaches. This article aims to critically review the main biological properties of scaffold-free regenerative procedures in dentistry. The most impactful pros and cons of the exosomes, the leading role of hypoxia-based mesenchymal stem cells (MSCs), and the strategic use of heat shock proteins in regenerative dentistry will be highlighted and discussed in terms of the use of such tools in dental regeneration and repair.

## 1. Introduction

The translational applications of regenerative medicine may impact on several fields of medicine. In the past ten years, the relatively new field of regenerative dentistry has achieved several discoveries, such as biomimetic materials, new orally derived stem cells, or interesting methods to populate scaffolds with cells and bioactive factors [1,2,3,4,5,6,7,8]. Typically, biomaterials can be synthetic or derived from several homologous and heterologous biological tissues. Mesenchymal stem cells (MSCs) are the main players of tissue regeneration; in the case of large bone defects, MSCs can be combined with scaffolds, achieving reparative or reconstructive results that are more effective than the physiological healing process [9,10].

Regenerative medicine procedures consist of surgical protocols that improve both the healing time and quality. The use of bioscaffolds in a surgical site can drive local stem cells towards the site in order to regenerate the surrounding tissue, while also enabling improved differentiation between specific phenotypes [11,12,13].

Several clinical applications of tissue engineering are well reported in the scientific literature. Studies on bone regeneration have combined mesenchymal stem cells derived from bone marrow and bioscaffolds, comparing such constructs with titanium mesh alone in the treatment of defects affecting the mandibular bone [13]. Furthermore, the use of adipose stem cells (ASCs) as scaffolds in micro-vascularized flaps has shown promising results in the treatment of bone defects [14].

A revolution is underway in the field of new materials; in fact, biomaterials have always been used to perform specific anatomical functions; however, today, the trend is to integrate their function within the material itself, thus developing scaffolds made up of multifunctional materials that can ‘feel the surrounding tissue’ and ‘react to it’ [11,12]. In the past, inert materials were used to rebuild or replace an organ; these were mostly metals, such as surgical steel. Over time, they have been overcome by materials that have mechanical, chemical and structural properties that can interact with the human body and surrounding tissues. Recently, biomaterial has also undergone development in manufacturing, with the introduction of technologies such as rapid prototyping or 3D printing, which have enabled the engineering and customization of grafting materials [13,14,15].

**Figure 1:**
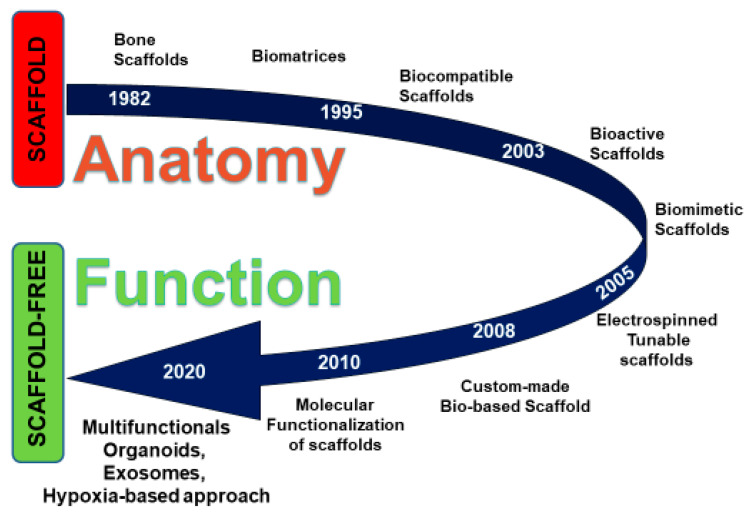
Time-dependent scheme representing the evolution of scaffold-based approaches and the recent development of scaffold-free methodologies.

The recent literature has, therefore, confirmed that the combination of MSCs, biomaterials and local growth factors can undoubtedly support the regeneration/repair of three-dimensional structures in different anatomic areas [15,16]. (Figure 1).

Recently, a novel intriguing approach was hypothesized: scaffold-free tissue engineering. We aim to briefly and critically discuss the most reliable, promising and predictable techniques of scaffold-free tissue regeneration and compare them to traditional techniques.

## 2. Searching Strategy

The first articles reporting the term “scaffold” in the scientific literature were published in the early 1960s. Then, the primary interest in scaffold applications was in relation to bone reconstruction. However, the term scaffold underwent several changes in the period between 1960 and 1980. In our narrative critical review, we have analysed the impact of the keyword “scaffold” in the title, in the abstract, and in the main text of articles published in PubMED-indexed journals in the timespan ranging from 1980 to 2020. Dental tissue engineering has pointed out a number of useful and smart alternatives to the classic concept of a scaffold; we considered the combination of terms such as “Platelets concentrates”, “Extracellular Matrix”, “Stem Cells”, “Proteins” and “Exosomes”. The main task in this preliminary step was to evaluate whether the topic discussed in these articles was relevant or not to our main aim, and to critically discuss such items alone and in comparison with each other.

When searching for the above-reported keywords in PubMed, we found 2215 items reported. Only highly impacted or well-recognized journals were considered in our research, as they represent a reliable and balanced selection of the most influential authors. Case reports, editorials, letters to editors, technical notes and brief reviews were not included in our overview.

The authors screened the title and abstract of the first selected articles and, after the exclusion of articles based on misleading topics, 173 papers related to the topic were considered and included in our overview.

First, we established the main objective of the work and the field (dentistry) considered in our study. In the second stage, we critically selected, read and compared the most interesting articles. Based on the expertise of the authors, we decided to select only the most relevant outcomes.

After the removal of duplicate articles, and other publication types, such as erratum and corrigendum, a total of 12 articles were selected for this review.

The main limitations of this study relate to the wide topic and the scarce observation time of the scaffold-free approaches, in comparison with the more predictable scaffolds, which have been well described and documented over the past forty years. Finally, the critical approach reported in this review is based on the authors’ expertise and insights into the matter, as the topics are substantially incomparable.

## 3. Scaffold-Free Approaches

### 3.1. Exosomes in Regenerative Dentistry

The scientific evidence shows the strong immunomodulatory activity of mesenchymal stem cells. MSCs conduct paracrine secretory activities, which allow MSCs to induce pleiotropic effects, especially local immunomodulation [17]. In 1967, Wolf and colleagues characterized various types of extracellular vesicles (EVs): such vesicles were originally named “platelet dust” [18]. EVs are released into the local environment and are created from the membrane of the vast majority of cells. In fact, they are delimited by a phospholipidic bilayer, similar to the cell from which they have been generated. These small vesicles are able to carry cell-specific loads, typically affecting the activity of target cells. A number of crucial cell processes, including cell-to-cell communication and the modulation of immune responses, are based on EV activity. The diameter of EVs is relevant for their characterization and they are divided into the following categories: (a) apoptotic bodies (>1000 nm), (b) microvesicles (100–1000 nm) and (c) exosomes (30–150 nm) [19]. It is commonly accepted that MSC-derived exosomes are one of the key paracrine effectors, through which MSCs can modulate tissue repair. The therapeutic effect of MSC-based therapy is largely caused by the local release of exosomes, which deliver specific molecules (proteins, nucleic acids, and lipids) to the local cells and tissues. Several intracellular proteins, such as mRNA, miRNA, and lipids, are carried by exosomes [20]. These components can regulate tissue formation by inhibiting or promoting specific pathways, according to the variable biology of the target site. In this landscape, exosome-based therapy can be applied to treat several disorders. For example, Joo et al. have shown the therapeutic effects of exosomes in heart, kidney, lung, skin, muscle, and brain diseases [21].

In the field of dental medicine, bone tissue regeneration is a fundamental process involved in the majority of therapies [14]. Angiogenesis plays a significant role in bone tissue repair, contributing to both bone growth and bone graft survival. Exosomes have been reported to induce local angiogenesis in bone regeneration: specifically, oral-derived stem cells are able to secrete exosomes loaded with pro-angiogenic miRNAs and direct them towards the local endothelial cells. Moreover, these small vesicles carry several biomolecules that possess signaling functions [18,19]. Petho et al. demonstrated that specific molecules carried by MSC-derived exosomes spread biochemical signals, promoting osteoblast differentiation and downregulating osteoclast formation [22].

Exosomes can be used as biomimetic tools, as they are able to trigger stem cell differentiation. Chun-Chieh et al. [23] highlighted how dental pulp stem cell (DPSC)-derived exosomes induce odontogenic differentiation in human dental pulp stem cells (DPSCs). Exosomes work by triggering strategic changes in MSCs, increasing odontogenic-related gene expression in vitro. Chew et al. investigated a ready-to-use and cell-free strategy for the treatment of periodontal defects in an immunocompetent rat model [24]. They used human MSC-derived exosomes and investigated their therapeutic effects on the healing process. The findings of this study suggest that exosome-based therapy can be applied to promote bone formation and periodontal regeneration. Based on such exciting results, the translational use—in several dental applications—of these nano-sized vesicles can be considered promising and worthy of being investigated further and in more depth by dentists in the coming years.

### 3.2. The Hypoxia-Based Approach in Regenerative Dentistry

Recent studies have highlighted the possibility of empowering the immunosuppressive, immunomodulatory and regenerative potential of stem cells through pre-conditioning techniques that work by using cytokines, hypoxia and other molecules [25]. Interestingly, the hypoxia-based approach has been investigated in several studies aimed to compare exosomes obtained from hypoxia-subjected MSCs, and exosomes obtained from normally cultured MSCs; the results show that the experimental protocol promoted angiogenesis in a significant way [26,27,28]. Moreover, microvesicles derived from hypoxia-subjected MSCs can influence the immune response, [29,30,31]. These immunomodulatory microparticles are able to both reduce local inflammation and support cell proliferation and migration. Regenerative dentistry may be supported by hypoxia-based MSC exosomes in the local induction of neo-angiogenesis [32,33]. In fact, newly formed tissues require strong vascularization to ensure the presence of nutrients and growth factors and to support cell vitality, proliferation and differentiation. In a recent review, Zimta et al. [34] summarized the available techniques to maximize the pro-angiogenic procedures applied in regenerative dentistry. In this review, the authors have focused on such micro-RNAs (miRNAs) that are able to influence the behavior of endothelial cells: these molecules will be used as pro-angiogenic factors in oral wound repair and regeneration [35]. Hypoxia-induced exosomes can be considered pro-angiogenic factors; essentially, these exosomes can stimulate the miR-135b target and hypoxia-inducible factor 1 (HIF-1). This peculiarity enhances angiogenesis, by positively regulating the expression of pro-angiogenic mediators acting in the oral tissues [35,36]. In conclusion, the biological condition of hypoxia appears to be strategic external stress, which is able to enhance the pro-angiogenic activity of conditioned MSCs by releasing bioactive exosomes.

### 3.3. Heat Shock Proteins (HSPs) in Regenerative Dentistry

Periodontal pathologies are one of the most common chronic inflammatory diseases affecting the oral cavity [37]. They are classified into different diagnostic categories [38], chronic periodontitis (CP) and acute periodontitis (AgP) [39]. In recent decades, many diagnostic and prognostic biomarkers have been proposed to treat periodontitis. Heat shock proteins (HSPs) seem to work well in promoting tissue regeneration [40] and are normally produced by cells exposed to stressful conditions. In 2010, Henderson et al. measured plasma levels of HSP10 and HSP60 in subjects with chronic periodontitis, and healthy subjects [41]. In this study, patients with chronic periodontitis showed significantly lower levels of HSP10, compared with healthy subjects; on the other hand, no significant difference was found in HSP60 levels. After a full periodontal treatment, the experimental group reported a significant increase in HSP10 levels, demonstrating the prognostic role of some circulating stress-related proteins as reliable indicators of successful periodontal treatments.

Heat shock protein 27 (HSP27), compared to HSP60, was investigated with the aim of understanding more about its ability to modulate intracellular responses to specific inflammatory signals by promoting anti-inflammatory pathways [42,43]. Blood levels of HSP27 seem to be related to different types of periodontitis, as assessed by Kaiser et al. [44]. The researchers found lower levels of HSP27 in a test group of patients suffering from aggressive periodontitis, compared to a second group involving patients suffering from chronic periodontitis and a third group involving only healthy subjects [45,46]. Furthermore, no differences were found in terms of HSP60 levels between the three groups described above. On the other hand, the study demonstrated a specific correlation between the levels of HSP27 in subjects with chronic periodontitis and healthy subjects. This evidence means that HSPs may play different roles in the modulation of different types of periodontitis. Specifically, HSP27 seems to be useful and appears to be involved in the regulation of the healing of acute periodontitis, as it increases in healthy subjects and in those subjects that have overcome the acute stage of periodontitis.

## 4. Conclusions and Future Insights

In our critical overview, we have pointed out the main pros and cons of both the approaches, with the most recent and promising literature sources (Table 1).

The micro- and nano-vesicles, such as the exosomes, have widely been demonstrated to be an effective approach, including through the use of the conditioned medium as a substitute for stem cells in the biological niche. Heat shock proteins (HSPs) and hypoxia-primed MSCs are also interesting, even if the subsequent continuous and uncontrolled/inhomogeneous stress to cell biology may trigger atypical replies from stem cells, producing anomalous clones and potential anomalous behavior by the regenerative niche.

Nowadays, no meaningful protocol for the in vivo application of a scaffold-free approach in translational dentistry exists. Future approaches may, of course, provide the proper combination of all the techniques reported here, and the authors cannot exclude the hypothesis that novel and smarter biomaterials may revolutionize the market of tissue engineering. As a matter of fact, the best approach does not exist at all: the only way to proceed is to analyze the clinical case closely and to use the best technique on a case-by-case basis. Generally and typically, large defects requiring wide replacements should always be performed with a scaffold-based approach; on the contrary, small lesions that are likely to be self-repairing may be treated with scaffold-free approaches to speed-up and improve the healing process.

## Figures and Tables

**Table 1:** Comparative table reporting the main scaffold-based and scaffold-free approaches in regenerative dentistry.

Study Model	Type of Disease	Therapeutic Approaches	Main Results	References
Fibrin sealant	Extraction wounds	Human plasma derivatives (Cell/scaffold-free)	Fibrin sealants enhance the healing process	Moller et al. 1988 [1]
Platelet-rich plasma (PRP)	Postoperative injury sites	Human plasma derivatives (Cell/scaffold-free)	The PRP delivers growth factors at the treated site	Whitman et al. 1997 [2]
Platelet-rich plasma (PRP)	Maxillary sinus-lift	Human plasma derivatives (Cell/scaffold-free)	Improvement in implant-prosthetic rehabilitation	Inchingolo et al. 2012 [3]
Platelet-rich fibrin (PRF)	Posto-operative surgical sites	Human plasma derivatives (Cell/scaffold-free)	PRF appears to accelerate the physiologic healing process	Dohan et al. 2006 [6]
Platelet-rich fibrin (PRF)	Post-extractive dental implants	Human plasma derivatives (Cell/scaffold-free)	Improving the bone quality and the healing of soft tissues	Marrelli et al. 2013 [7]
DPSCs + hydrogel scaffolds (bECM) + GFs	In vitro experiments	Cell/scaffold/GFs	Up-regulation of osteogenic genes	Paduano et al. 2016 [9]
Cell sheets engineering (CSE)	In vitro experiments	Scaffold-free cell sheet engineering (CSE)	Improving of cell growth to improve the viability of the cell grafts	Moschouris et al. 2016 [12]
The microvascular custom-made ectopic bone flap	Hemi-maxillectomy	Cell/scaffold/GFs	Reconstruction of large defects	Mesimaki et al. 2009 [14]
DPSCs + Exosomes	In vitro experiments	Cell/biomolecules	Culture and growth of dental pulp-like tissue in a tooth-root model	Chun-Chieh et al. 2016 [23]
MSC-derived exosome-loaded collagen sponge	Periodontal defects	Cell/scaffold/biomolecules	Periodontal tissue regeneration through increased cellular mobilization and proliferation	Chew et al. 2019 [24]
Exosomes from hypoxia-cultured MSCs	In vitro experiments	Cell/biomolecules	Anti-inflammatory activity	Showalter et al. 2019 [31]
Exosomes-derived miRNA	In vitro experiments	Cell/biomolecules	Pro-angiogenic activity	Zimta et al. 2019 [34]

## References

[1] Moller J.F., Petersen J.K. (1988). Efficacy of a fibrin sealant on healing of extraction wounds. Int. J. Oral Maxillofac. Surg..

[2] Whitman D.H., Berry R.L., Green D.M. (1997). Platelet gel: An autologous alternative to fibrin glue with applications in oral and maxillofacial surgery. J. Oral Maxillofac. Surg..

[3] Inchingolo F., Tatullo M., Marrelli M., Inchingolo A.M., Inchingolo A.D., Dipalma G., Flace P., Girolamo F., Tarullo A., Laino L. (2012). Regenerative surgery performed with platelet-rich plasma used in sinus lift elevation before dental implant surgery: An useful aid in healing and regeneration of bone tissue. Eur. Rev. Med. Pharmacol. Sci..

[4] Choukroun J., Adda F., Schoeffler C., Vervelle A. (2001). Une opportunité en paro-implantologie: Le PRF. Implantodontie.

[5] Shah R., M.G.T., Thomas R., Mehta D.S. (2017). An Update on the Protocols and Biologic Actions of Platelet Rich Fibrin in Dentistry. Eur. J. Prosthodont. Restor. Dent..

[6] Dohan D.M., Choukroun J., Diss A., Dohan S.L., Dohan A.J., Mouhyi J., Gogly B. (2006). Platelet-rich fibrin (PRF): A second-generation platelet concentrate. Part III: Leucocyte activation: A new feature for platelet concentrates?. Oral Surg. Oral Med. Oral Pathol. Oral Radiol. Endod..

[7] Marrelli M., Tatullo M. (2013). Influence of PRF in the healing of bone and gingival tissues. Clinical and histological evaluations. Eur. Rev. Med. Pharmacol. Sci..

[8] Barry M., Pearce H., Cross L., Tatullo M., Gaharwar A.K. (2016). Advances in Nanotechnology for the Treatment of Osteoporosis. Curr. Osteoporos. Rep..

[9] Paduano F., Marrelli M., White L.J., Shakesheff K.M., Tatullo M. (2016). Odontogenic Differentiation of Human Dental Pulp Stem Cells on Hydrogel Scaffolds Derived from Decellularized Bone Extracellular Matrix and Collagen Type, I. PLoS ONE.

[10] Marrelli M., Maletta C., Inchingolo F., Alfano M., Tatullo M. (2013). Three-point bending tests of zirconia core/veneer ceramics for dental restorations. Int J Dent.

[11] Tatullo M., Codispoti B., Pacifici A., Palmieri F., Marrelli M., Pacifici L., Paduano F. (2017). Potential use of human periapical cyst-mesenchymal stem cells (hpcy-mscs) as a novel stem cell source for regenerative medicine applications. Front. Cell Dev. Biol..

[12] Moschouris K., Firoozi N., Kang Y. (2016). The application of cell sheet engineering in the vascularization of tissue regeneration. Regen. Med..

[13] Warnke P.H., Wiltfang J., Springer I., Acil Y., Bolte H., Kosmahl M., Russo P.A.J., Sherry E., Lutzen U., Wolfart S. (2006). Man as living bioreactor: Fate of an exogenously prepared customized tissue-engineered mandible. Biomaterials.

[14] Mesimaki K., Lindroos B., Tornwall J., Mauno J., Lindqvist C., Kontio R., Miettinen S., Suuronen R. (2009). Novel maxillary reconstruction with ectopic bone formation by GMP adipose stem cells. Int. J. Oral Maxillofac. Surg..

[15] Kerativitayanan P., Tatullo M., Khariton M., Joshi P., Perniconi B., Gaharwar A.K. (2017). Nanoengineered Osteoinductive and Elastomeric Scaffolds for Bone Tissue Engineering. ACS Biomater. Sci. Eng..

[16] Langer R., Vacanti J.P. (1993). Tissue engineering. Science.

[17] Zhou Y., Yamamoto Y., Xiao Z., Ochiya T. (2019). The Immunomodulatory Functions of Mesenchymal Stromal/Stem Cells Mediated via Paracrine Activity. J. Clin. Med..

[18] Wolf P. (1967). The nature and significance of platelet products in human plasma. Br. J. Haematol..

[19] Gurunathan S., Kang M.H., Jeyaraj M., Qasim M., Kim J.H. (2019). Review of the Isolation, Characterization, Biological Function, and Multifarious Therapeutic Approaches of Exosomes. Cells.

[20] Lai R.C., Arslan F., Lee M.M., Sze N.S.K., Choo A., Chen T.S., Saltotellez M., Timmers L., Lee C.N., el Oakley R.M. (2010). Exosome secreted by MSC reduces myocardial ischemia/reperfusion injury. Stem Cell Res..

[21] Joo H.S., Suh J.H., Lee H.J., Bang E.S., Lee J.M. (2020). Current Knowledge and Future Perspectives on Mesenchymal Stem Cell-Derived Exosomes as a New Therapeutic Agent. Int. J. Mol. Sci..

[22] Pethő A., Chen Y., George A. (2018). Exosomes in Extracellular Matrix Bone Biology. Curr. Osteoporos. Rep..

[23] Chun-Chieh H., Raghuvaran N., Satish A., Sriram R. (2016). Exosomes as biomimetic tools for stem cell differentiation: Applications in dental pulp tissue re generation. Biomaterials.

[24] Chew J.R.J., Chuah S.J., Teo K.Y.W., Zhang S., Lai R.C., Fu. J.H., Lim L.P., Lim S.K., Toh W.S. (2019). Mesenchymal stem cell exosomes enhance periodontal ligament cell functions and promote periodontal regeneration. Acta Biomater..

[25] Noronha N.C., Mizukami A., Caliari-Oliveira C., Cominal J.G., Rocha J.L.M., Covas D.T., Swiech K., Malmegrim K.C.R. (2019). Priming approaches to improve the efficacy of mesenchymal stromal cell-based therapies. Stem Cell Res. Ther..

[26] Zhang H.C., Liu X.B., Huang S., Bi X.Y., Wang H.X., Xie L.X., Wang Y., Cao X., Lv J., Xiao F. (2012). Microvesicles derived from human umbilical cord mesenchymal stem cells stimulated by hypoxia promote angiogenesis both in vitro and in vivo. Stem Cells Dev..

[27] Salomon C., Ryan J., Sobrevia L., Kobayashi M., Ashman K., Mitchell M., Rice G.E. (2013). Exosomal signaling during hypoxia mediates microvascular endothelial cell migration and vasculogenesis. PLoS ONE.

[28] Han Y.D., Bai Y., Yan X.L., Ren J., Zeng Q., Li X.D., Pei X.T., Han Y. (2018). Co-transplantation of exosomes derived from hypoxia-preconditioned adipose mesenchymal stem cells promotes neovascularization and graft survival in fat grafting. Biochem. Biophys. Res. Commun..

[29] Lee S.C., Jeong H.J., Lee S.K., Kim S.J. (2016). Hypoxic Conditioned Medium from Human Adipose-Derived Stem Cells Promotes Mouse Liver Regeneration Through JAK/STAT3 Signaling. Stem Cells Transl. Med..

[30] Lin S., Zhu B., Huang G., Zeng Q., Wang C. (2019). Microvesicles derived from human bone marrow mesenchymal stem cells promote U2OS cell growth under hypoxia: The role of PI3K/AKT and HIF-1alpha. Hum. Cell.

[31] Showalter M.R., Wancewicz B., Fiehn O., Archard J.A., Clayton S., Wagner J., Deng P., Halmai J., Fink K.D., Bauer G. (2019). Primed mesenchymal stem cells package exosomes with metabolites associated with immunomodulation. Biochem. Biophys. Res. Commun..

[32] Riccitiello F., De Luise A., Conte R., D’Aniello S., Vittoria V., Di Salle A., Calarco A., Peluso G. (2018). Effect of resveratrol release kinetic from electrospun nanofibers on osteoblast and osteoclast differentiation. Eur. Polym. J..

[33] Saghiri M.A., Asatourian A., Sorenson C.M., Sheibani N. (2015). Role of angiogenesis in endodontics: Contributions of stem cells and proangiogenic and antiangiogenic factors to dental pulp regeneration. J. Endod..

[34] Zimta A.A., Baru O., Badea M., Buduru S.D., Berindan-Neagoe I. (2019). The Role of Angiogenesis and Pro-Angiogenic Exosomes in Regenerative Dentistry. Int. J. Mol. Sci..

[35] Bian S., Zhang L., Duan L., Wang X., Min Y., Yu H. (2014). Extracellular vesicles derived from human bone marrow mesenchymal stem cells promote angiogenesis in a rat myocardial infarction model. J. Mol. Med..

[36] Fan G.C. (2014). Hypoxic exosomes promote angiogenesis. Blood.

[37] Eke P.I., Dye B.A., Wei L., Thorntonevans G., Genco R.J. (2012). Prevalence of periodontitis in adults in the United States: 2009 and 2010. J. Dent. Res..

[38] Armitage G.C. (2004). Periodontal diagnoses and classification of periodontal diseases. Periodontology 2000.

[39] Inchingolo F., Tatullo M., Marrelli M., Inchingolo A.M., Picciariello V., Inchingolo A.D., Dipalma G., Vermesan D., Cagiano R. (2010). Clinical trial with bromelain in third molar exodontia. Eur. Rev. Med. Pharmacol. Sci..

[40] Henderson B., Pockley A.G. (2012). Proteotoxic stress and circulating cell stress proteins in the cardiovascular diseases. Cell Stress Chaperones.

[41] Tatullo M., Marrelli M., Scacco S., Lorusso M., Doria S., Sabatini R., Auteri P., Cagiano R., Inchingolo F. (2012). Relationship between oxidative stress and “burning mouth syndrome” in female patients: A scientific hypothesis. Eur. Rev. Med. Pharmacol. Sci..

[42] De A.K., Kodys K.M., Yeh B.S., Miller-Graziano C. (2000). Exaggerated human monocyte IL-10 concomitant to minimal TNF-alpha induction by heat-shock protein 27 (Hsp27) suggests Hsp27 is primarily an antiinflammatory stimulus. J. Immunol..

[43] Laudanski K., De A., Miller-Graziano C. (2007). Exogenous heat shock protein 27 uniquely blocks differentiation of monocytes to dendritic cells. Eur. J. Immunol..

[44] Kaiser F., Donos N., Henderson B., Alagarswamy R., Pelekos G., Boniface D., Nibali L. (2018). Association between circulating levels of heat-shock protein 27 and aggressive periodontitis. Cell Stress Chaperones.

[45] Ballini A., Cantore S., Scacco S., Coletti D., Tatullo M. (2018). Mesenchymal Stem Cells as Promoters, Enhancers, and Playmakers of the Translational Regenerative Medicine 2018. Stem Cells Int..

[46] Marrazzo P., Paduano F., Palmieri F., Marrelli M., Tatullo M. (2016). Highly Efficient In Vitro Reparative Behaviour of Dental Pulp Stem Cells Cultured With Standardised Platelet Lysate Supplementation. Stem Cells Int..

